# Artificial Intelligence-Based Classification of Chest X-Ray Images into COVID-19 and Other Infectious Diseases

**DOI:** 10.1155/2020/8889023

**Published:** 2020-10-06

**Authors:** Arun Sharma, Sheeba Rani, Dinesh Gupta

**Affiliations:** Translational Bioinformatics Group, International Centre for Genetic Engineering and Biotechnology (ICGEB), Aruna Asaf Ali Marg, New Delhi 110067, India

## Abstract

The ongoing pandemic of coronavirus disease 2019 (COVID-19) has led to global health and healthcare crisis, apart from the tremendous socioeconomic effects. One of the significant challenges in this crisis is to identify and monitor the COVID-19 patients quickly and efficiently to facilitate timely decisions for their treatment, monitoring, and management. Research efforts are on to develop less time-consuming methods to replace or to supplement RT-PCR-based methods. The present study is aimed at creating efficient deep learning models, trained with chest X-ray images, for rapid screening of COVID-19 patients. We used publicly available PA chest X-ray images of adult COVID-19 patients for the development of Artificial Intelligence (AI)-based classification models for COVID-19 and other major infectious diseases. To increase the dataset size and develop generalized models, we performed 25 different types of augmentations on the original images. Furthermore, we utilized the transfer learning approach for the training and testing of the classification models. The combination of two best-performing models (each trained on 286 images, rotated through 120° or 140° angle) displayed the highest prediction accuracy for normal, COVID-19, non-COVID-19, pneumonia, and tuberculosis images. AI-based classification models trained through the transfer learning approach can efficiently classify the chest X-ray images representing studied diseases. Our method is more efficient than previously published methods. It is one step ahead towards the implementation of AI-based methods for classification problems in biomedical imaging related to COVID-19.

## 1. Introduction

Coronavirus disease 2019 (COVID-19) is an infectious disease triggered by severe acute respiratory syndrome coronavirus 2 (SARS-CoV-2) [1]. The disease was initially identified in December 2019 in Wuhan, China, and has since spread globally [2, 3]. At the outset, a patient with pneumonia of mysterious cause was first reported to the WHO Country Office in China on 31 December 2019 [4]. Since then, the disease has spread all over the globe in enormous numbers and is declared a pandemic. As of 16 September 2020, there were 29356292 confirmed COVID-19 cases in various countries, territories, or areas, and 930260 people had lost their lives [5], and the numbers are still rising. Although radiological imaging is not recommended for diagnostics as the patient arrives in the clinic, a chest X-ray is often useful to monitor treatment outcomes and comorbidities in seriously ill patients. The detection of COVID-19 from chest X-ray and its differentiation from lung diseases with identical opacities is a puzzling task that relies on the availability of expert radiologists.

Recently, several researchers have reported the use of AI-based tools in solving image classification problems in healthcare, based on training with X-ray images, CT scans, histopathology images, etc. Deep learning is an extremely powerful tool for learning complex, cognitive problems [6, 7], and the frequency of their use and evaluation in different problems is increasing [8]. In the present study, we have made use of a deep learning algorithm using the convolutional neural network (CNN) that can efficiently detect COVID-19 from chest X-ray images for swift diagnosis.

Due to data scarcity related to COVID-19 chest X-ray images, instead of training the model from scratch, the present study made use of the “transfer learning method” by leveraging the already available models in solving the analogous problems [9–11]. Moreover, transfer learning eases the hypothesis that the training data must be independent and identically distributed with the test data [12].

Recently, an attempt made to detect the novel coronavirus using CT images, employing a deep learning algorithm, achieved an internal validation accuracy of 82.9% and external validation accuracy of 73.1% [13]. Another study performed by Xu et al. established a screening model to distinguish COVID-19 pneumonia from influenza A viral pneumonia and healthy cases with pulmonary CT images. It achieved an accuracy of 86.7% for the benchmark dataset [14]. Zheng et al. achieved 90.1% accuracy (using a probability threshold of 0.5) using the CT images for the detection of the COVID-19 [15]. Notably, quite a few research groups also report the development of deep learning or AI-based classification models for COVID-19 based on chest X-ray images [16–21].

For example, Asnaoui et al. achieved 92.18% accuracy (for Inception-ResNetV2) to classify the chest X-ray and CT images into bacterial pneumonia, coronavirus, and normal classes [16]. Ozturk et al. developed deep learning-based binary classification (COVID vs. no findings) and multiclass classification (COVID vs. no findings vs. pneumonia) models that achieved the highest accuracies of 98.08% and 87.02%, respectively [17]. Waheed et al. developed a method to generate synthetic chest X-ray (CXR) images by creating an Auxiliary Classifier Generative Adversarial Network- (ACGAN) based model CovidGAN. The binary classification models achieved an accuracy of 85% for the model based on original images (training dataset consisted of 331 COVID-CXR images and 601 normal-CXR images). However, the accuracy increased to 95% for the model trained with the combined use of original and augmented images (training dataset consisting of actual images plus CovidGAN generated 1399 synthetic images of normal-CXR and 1669 synthetic COVID-CXR images). Thus, the original image-based dataset consisted of 932 training samples (331 COVID-CXR and 601 normal-CXR images). In comparison, the combined dataset of original and synthetic images consisted of 4000 training samples (2000 COVID-CXR and 2000 normal-CXR images). They evaluated performance on 192 testing samples for the two models—namely, the model trained with original and the one with the original as well as synthetic images [18]. Chouhan et al. developed a transfer learning-based approach for the prediction of paediatric pneumonia based on chest X-ray images. The ensemble model developed in the study achieved a maximum accuracy of 96.4% with a recall of 99.62% on unseen data [22]. Jaiswal et al. developed a deep learning-based approach (using Mask-RCNN) for the identification and localization of pneumonia in chest X-ray (CXR) images. The study used three types of chest X-ray images, viz., lung opacity, abnormal, and normal, for training and testing the deep learning models [23]. Che Azemin et al. developed a deep learning-based COVID-19 prediction model using publicly available radiologist-adjudicated chest X-ray images. The binary classification model classified the test chest X-ray images into COVID-19 and none with maximum values of 0.82, 77.3%, 71.8%, and 71.9% for the area under the receiver operating curve, sensitivity, specificity, and accuracy, respectively [24]. However, the criteria used to select the COVID-19 images (for training the deep learning models) seem to be questionable.

Ucar and Korkmaz developed COVIDiagnosis-Net (based on deep SqueezeNet with Bayes optimization) for the diagnosis of COVID-19 with an overall test accuracy of 98.26%. The method classifies the three-class X-ray images labeled as normal (no infection), pneumonia (bacterial or non-COVID viral infection), and COVID (COVID-19 viral infection) [19]. Oh et al. statistically analyzed the potential chest X-ray (CXR) COVID-19 markers to understand the statistically significant differences. The various parameters studied by them include lung morphology, mean lung intensity, and the standard deviation of lung intensity in normal lungs, bacterial pneumonia, viral pneumonia, tuberculosis, and COVID-19 CXR images. The model achieved an overall highest classification accuracy of 88.9%, using a local patch-based approach for the four different classes [20]. Apostolopoulos et al. trained convolutional neural networks (CNNs) from scratch and achieved 87.66% classification accuracy between the seven classes (normal, pneumonia, COVID-19, pulmonary edema, pleural effusion, Chronic Obstructive Pulmonary Disease (COPD), and pulmonary fibrosis). In the case of binary classification (COVID-19 vs. non-COVID-19), a maximum of 99.18% accuracy, 97.36% sensitivity, and 99.42% specificity was achieved [21]. Pereira et al. developed multiclass and hierarchical learners that achieved a macro-avg F1-score of 0.65 and 0.89, respectively, for COVID-19 identification in chest X-ray images [25]. Rahimzadeh and Attar developed multiclass (normal vs. pneumonia vs. COVID-19) classification methods. They achieved the highest average accuracy of 99.50% and overall average accuracy of 91.4% for COVID-19 cases and all the three classes, respectively [26]. Recent reports indicate that efforts are on to develop better COVID-19 chest X-ray classification models with a large number of images [27–30]. Although the models claim to possess high classification accuracies, these are devoid of systematic approaches—including proper data preprocessing (a vital step during the model training and validation) and use of comprehensive augmentation techniques (an essential requirement for the development of generalized models). The lacunae in the recently published studies motivated us to develop a better classification method, addressing the shortcomings.

The present study is the first to use a few COVID-19 chest X-ray images to train the classification models, while almost double image dataset for external or independent validation of the developed models. One of the uniqueness of the present study is the age-based selection criteria of chest X-ray images for the training and testing of AI-based models. None of the studies used age as a selection criterion (during the preparation of image datasets); thus, paediatric images have been classified (as a separate class, i.e., pneumonia vs. normal vs. COVID-19) from the adult group images. This may lead to biased learning of the deep learning-based models. For example, a model that has been trained on paediatric age group “pneumonia” and “normal” chest X-ray images, and adult age group “COVID-19” images, will have a greater probability of classifying unknown adult age group images as “COVID-19,” while paediatric age group images as “pneumonia” or “normal.” Moreover, the images used in our study are downloaded from the sources containing unique images to avoid any duplication of training and testing set images. In contrast, many studies claiming a large number of COVID-19 images (downloaded from multiple publicly available sources) in actual might possess duplicate images, which might be affecting the prediction results. For the majority of the studies discussed above, neither the classification models nor the codes used to train and evaluate the models are publicly shared. However, the codes (used to train and validate the models), validation datasets, and the models developed in the present study are publicly available to assist the scientific community for further development in the area of chest X-ray-based COVID-19 or other infectious diseases classification. Thus, the results have been encouraging and ensure high prediction accuracy in real-life cases, as evident from the high prediction accuracies (of prediction the models) on external validation datasets. The study reports the use of transfer learning with the highest number of image augmentation types (25 different types) utilized for the development of high-classification accuracy AI-based chest X-ray classification models. The models, deployable as a web server, can rapidly classify input posteroanterior (PA) chest X-ray images into images corresponding to COVID-19, pneumonia, TB, non-COVID-19, and normal subjects.

## 2. Materials and Methods

### 2.1. Source of Chest X-Ray Images

For the training and development of AI-based classification models, COVID-19, non-COVID-19, pneumonia, tuberculosis (TB), and normal chest X-ray images were downloaded from three different sources as given in Table S1. During the development of classification models and preparation of the manuscript for the present study, new images were added to the COVID-19 images of GitHub resource. The images, downloaded on May 29, 2020, were filtered on the criteria such as age > 18 years, PA view, and COVID-19-only chest X-ray images to retrieve the images of interest. Thus, a total of 75 COVID-19 chest X-ray images were used as “external validation dataset-II” for better and rigorous evaluation of the classification models. Henceforth, these images are referred to as “New COVID-19.”

### 2.2. Selection of Images for the Study

The downloaded images were manually curated to filter out a similar type of images and retain only the posteroanterior view chest X-ray images of adults. Images with no information about the patient's age or age < 19 years, chest X-ray view other than posteroanterior (PA), and CT images were excluded (Tables S2–S6) from the training dataset. Post-selection, a total of 352 chest X-ray images (original images) were left for training and testing of the AI-based models (Table 1). Further, we distributed the original images into 51, 21, 160, 54, and 66 images for COVID-19, non-COVID-19, pneumonia, TB, and normal, respectively.

### 2.3. Dataset Preparation

**Figure 1:**
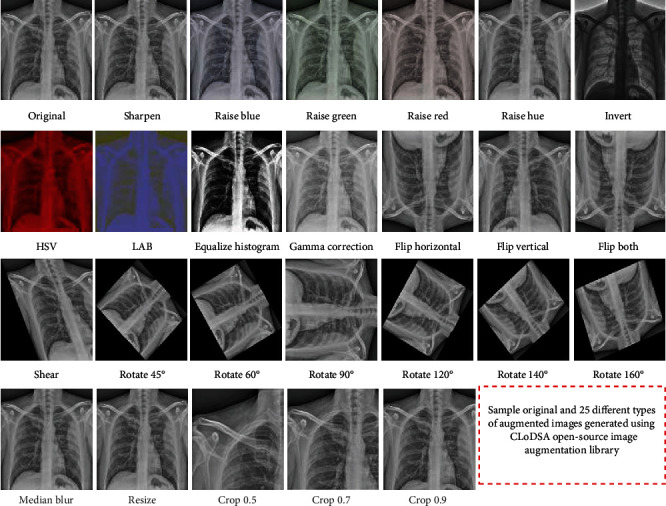
Sample original and 25 different types of augmented images (generated through open-source, image augmentation library—CLoDSA).

**Figure 2:**
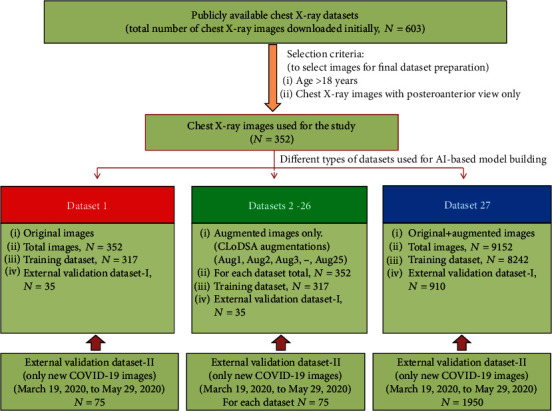
The methodology used for the preparation of datasets for model training and evaluation.

For the training and testing of AI-based models (Table 1), the original image dataset was divided into 90% training dataset (317 images) and 10% external validation dataset-I (35 images). As the number of images was limited, we generated 25 different types of augmentations (Figure 1) through an open-source augmentation tool CLoDSA [31] (Figure 2, Table S7). Thus, a total of 27 different types of training, external validation datasets I and II, for chest X-ray images were generated using JSON scripts [32].

**Figure 3:**
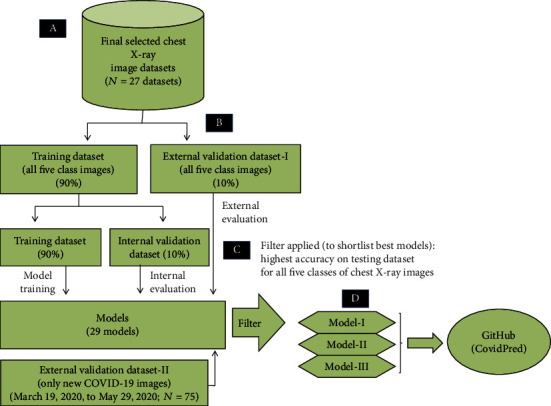
Flowchart depicting the methodology used for the training, evaluation, validation, and selection of AI-based models available on the GitHub link.

Out of the 27 datasets, one dataset comprised of original images (dataset 1), and 25 datasets (datasets 2-26) consist of single augmentation images, while the combination of the former 26 different types of datasets generated a combined dataset (dataset 27). All the 27 different types of datasets were used to train and validate the 29 different types of AI-based chest X-ray classification models. The original and single augmentation-based models were trained and tested/validated using 317 and 35 images, respectively (Figure 2, Table S7). The combined dataset used 8242 and 910 images for training and external validation of AI-based models, respectively (Figure 1, Table S7). Furthermore, for the hyperparameter optimization and internal validation of AI-based models, all the 27 training datasets were further split into 90% training datasets and 10% internal validation datasets (Figure 3). Additionally, we used external validation dataset-II to evaluate the performance of all the 29 models for COVID-19 chest X-ray images.

### 2.4. Techniques Used

We used the transfer learning approach to train and validate the 29 (27 plus, dataset 27 trained with two additional epoch sizes) different types of AI-based models (Figure 3). The Python scripts used to train and validate the models are available at https://github.com/arunsharma8osdd/covidpred. The hyperparameters used to train the models are given in Table 2.

A total of 29 different types of models were trained and validated, making use of 27 datasets (Figure 3). Out of the 29 models, one model was trained and validated on dataset 1, while 25 models made use of datasets 2-26. All the 26 models were trained on 24 epochs, with the hyperparameter values (Table 2(a)). The higher number of epochs was avoided to prevent overfitting of the models. The remaining three models made use of dataset 27 with varying numbers of iterations and epochs. These 24, 49, and 101 epoch-based models used 5568, 11136, and 23300 iterations, respectively.  Table 2(b) provides the hyperparameter values used by these three models. The models showing the highest accuracy on external validation datasets were selected as best-performing models and uploaded to the project GitHub page (named “CovidPred”).

## 3. Results

### 3.1. Performance of Original Image-Based Model

The AI-based models were trained and validated using original images and external validation datasets containing 317 and 35 chest X-ray images, respectively. Moreover, to evaluate the real-life performance of models (especially for COVID-19 images), external validation dataset-II was used. For tuning the hyperparameters (during the training process), the training dataset was further divided into a 90% training set (286 images) and a 10% internal validation dataset (31 images). A maximum training and internal validation accuracy (%) of 100 and 75 were achieved for the training and internal validation datasets, respectively. Further evaluation of the trained models using external validation dataset-I revealed accuracies (%) of 57.14, 80, 53.33, 50, 68.75, and 60 for normal, COVID-19, new COVID-19, non-COVID-19, pneumonia, and tuberculosis, respectively (Table S8). Thus, a lower accuracy (%) of 53.33 is achieved for the “new COVID-19 images” (external validation dataset-II images).

### 3.2. Performance of Single Augmentation-Based Models

Training and testing were performed for 25 datasets, using different types of training and validation/testing dataset images (Table S8). Each dataset was trained with 286 images, and evaluation was performed using 31 internal validation and 110 external validation dataset images. As evident from Table 3, using the model based on 120° rotated images, maximum training and internal validation accuracies (%) of 100 and 62 were achieved for training and internal validation datasets, respectively. Furthermore, the highest accuracies (%) of 14.29, 20, 14.67, 100, 100, and 100 are achieved on the testing dataset for normal, COVID-19, new COVID-19, non-COVID-19, pneumonia, and tuberculosis, respectively (Table 3, Figure S1).

Using the model based on 140° rotated images, maximum training and internal validation accuracies (%) of 100 and 81.2 were achieved for training and internal validation datasets, respectively. The highest accuracies (%) of 100, 100, 94.67, 0, 93.75, and 0 were achieved on the testing dataset for normal, COVID-19, new COVID-19, non-COVID-19, pneumonia, and tuberculosis, respectively (Table 3, Figure S2). These two models were considered as best-performing complementary models. Thus, the selection of two models ensured that poor performance for one type of image shown by one model could be overcome by the second model and vice versa.

### 3.3. Performance of Original Images and Augmentation (Combined Dataset)-Based Models

The combined image-based model contained 7418, 824, 910, and 1950 images in the training dataset, internal validation dataset, external validation dataset-I, and external validation dataset-II, respectively. The 24 epoch-based model achieved maximum training and internal validation accuracies (%) of 100 and 93.8, for training and internal validation datasets, respectively. Further, evaluation using combined testing dataset images showed the highest accuracies (%) of 73.63, 56.92, 42.62, 53.85, 96.39, and 75.38, for normal, COVID-19, new COVID-19, non-COVID-19, pneumonia, and Tuberculosis, respectively (Table 4).

For the 49 epoch-based model, the maximum training and internal validation accuracies (%) of 100 was achieved for both the training and the internal validation datasets. Further, evaluation using combined testing dataset images showed the highest accuracies (%) of 86.26, 65.38, 43.59, 42.31, 96.15, and 73.08, for normal, COVID-19, new COVID-19, non-COVID-19, pneumonia, and tuberculosis, respectively (Table 4).

The 101 epoch-based model achieved maximum training and internal validation accuracies (%) of 100 and 93.8, for training and internal validation datasets, respectively. Further, evaluation using combined testing dataset images showed the highest accuracies (%) of 85.71, 70.77, 51.28, 51.92, 93.99, and 74.62, for normal, COVID-19, new COVID-19, non-COVID-19, pneumonia, and tuberculosis, respectively (Table 4).

Thus, none of the combined dataset-based three models achieved the desirable high accuracies on the combined testing dataset for all the five types of image classes. Therefore, in-depth analysis or classification was performed (by supplying the single kind of augmentation-based images at a time) to determine which types of images these models identify and classify with the highest accuracy (Table S9). The 101 epoch-based model, using rotate 60° images, achieved the highest accuracies (%) of 100, 100, 66.67, 100, 93.75, and 80, for normal, COVID-19, new COVID-19, non-COVID-19, pneumonia, and tuberculosis images, respectively (Table 5, Figure S3). Hence, the best-performing 101 epoch-based model is also selected as the third complementing model.

## 4. Discussion

The confirmatory diagnosis of COVID-19 is mainly dependent on clinical symptoms, epidemiological history, nucleic acid detection, immune identification technology, etc. All the methods mentioned above have some limitations such as time required, costs [13, 33], equipment dependence, shortage of testing kits [34], availability of trained healthcare workers, interoperator variabilities, especially in a pandemic like this, making them cumbersome diagnostic procedures [34]. The auxiliary examinations, for example, nucleic acid identification technologies, suffer from the false-negative rate, which cannot be overlooked [33]. In a situation of worldwide medical emergencies like the current COVID-19 pandemic, it is desirable to have a fast, cost-effective, user-friendly, noninvasive, and intelligent diagnostic method for rapid screening and early diagnosis of diseases, which also requires the least manual intervention. Timely diagnosis of the COVID-19 patients can enable help in the optimization of available resources, including trained human resources, for all the supportive measures required for confirmed patients. Automated AI-based intelligent chest X-ray classification has such untapped potential for this unmet need, as evident from recent researches. The most commonly used radiological diagnostic imaging is chest X-rays, as compared to computed tomography (CT) and magnetic resonance imaging (MRI), due to its low cost and less processing time and lower radiation exposure [35]. In pandemics, like the current one, it is crucial to quarantine suspected patients for their proper treatment quickly. Rapid screening to diagnose such patients is also essential for controlling outbreaks. AI-based disease classification may also be combined with confirmatory laboratory testing.

Similarly, it may be an excellent proposition to aid prognosis and evaluation of recuperating/follow-up patients, using AI-based rapid and less time-consuming tools. Recently, researchers have made attempts to develop chest X-ray image-based COVID-19 classification or identification methods [36], with different capabilities. However, the studies possess some significant limitations that need to be resolved to develop more reliable and accurate classification models. To mention a few deficiencies, few of the studies have included CXR images (in different classes) from highly divergent age groups, i.e., paediatric as well as adults [16, 19, 21]. The procedure makes the classification job easy (for deep learning algorithms), but at the same time, it is biased due to the differences in lung sizes of paediatric vs. adult age groups. The chances are there that new unknown images belonging to a particular age group will be automatically assigned to a class having images similar to that age group, instead of diseased vs. normal or any other criteria. The majority of studies [16–19, 21, 25, 26] did not apply the age group (paediatric only or adolescent only or adults only) and CXR view (PA or posteroanterior, LL or laterolateral, etc.) inclusion criteria. The absence of these kinds of selection criteria may lead to inappropriate training of models, and that may not perform well in real-life situations.

For the generalization of models and to increase the sizes of the datasets, only a few studies have made use of augmentation techniques with a minimal number of augmentation types [19, 21]. Moreover, few of the reviewed studies have used commercial software such as MATLAB for image analysis and deep learning-based algorithms or model development [19, 20]. The use of commercial software limits the wide usage of such studies at the user's end, easy deployment for free usage, and further development. Another study improperly merged a non-COVID-19 SARS patient's images into COVID-19 image class during the training of deep learning-based models [20]; this can badly affect the prediction outcomes of models, and thus, non-COVID-19 images may be classified as COVID-19. The latter may lead to high false positives during real-life predictions. Also, the most recent studies claiming high prediction accuracies have been tested/validated on small COVID-19 CXR image test datasets (almost 1/3 of the COVID-19 external validation dataset CXR images) in the present study [25, 26].

Our study design overcomes the limitations of recently published studies. Machine learning or AI-based training requires similar types of medical images (mainly captured from the same view and similar age group) for the development of efficient classification models. Therefore, manual curation of the publicly downloaded dataset helps to retain similar types of images (having a single type of view, i.e., posteroanterior, and age group). The augmentation of images has been a popular technique in the area of computer vision, commonly used to increase the dataset size and develop the generalized models [37–40]. Also, transfer learning approaches are useful to solve several image classification-based biomedical problems [41–43]. Therefore, in the present study, for the first time, successful attempts have been made to develop augmented images based on efficient classification models for COVID-19 and other infectious diseases. The transfer learning approach helped in saving a lot of time and effort required to develop highly accurate classification models, even using a minimal number of images. A total of three best-performing AI-based models trained and tested on (i) 120° rotated images, (ii) 140° rotated images, and (iii) a combination of original and 25 different types of augmented images have been provided on the GitHub.

Due to the availability of limited numbers of chest X-ray images for COVID-19, the AI-based models trained on fewer images have the chance of overfitting in the classification models. However, the performance of our models on independent datasets rules out any indication of overfitting of the models. In the future, the model training will be enhanced further by incorporating a large number of images to develop more robust and scalable classification models. The currently developed AI-based models in the study are CPU-based, hence slow in the classification of chest X-rays. In the future, we will focus on the development of GPU-based classification models that will help in providing bulk upload of image facility to users through a user-friendly web interface. Only chest X-rays with PA views were used in training the models; as of now, no other radiological images can be used for model evaluation.

As the chest X-ray data on COVID-19 is rapidly increasing, the authors are continuously putting efforts to make use of maximum available data to update the classification models and enhance their reliability and utility in the real-life situations. Moreover, the availability of more and diverse training images will facilitate the development of more robust and scalable classification models.

## 5. Conclusions

AI-based classification can help rapid diagnosis of COVID-19 and other major infectious diseases. The models developed by us are proof of the concept that cost-effective, user-friendly, and noninvasive AI-based methods can be developed for COVID-19. The AI-based models developed by us may be evaluated for its use in clinics, as diagnostic or clinical management of patients. Also, in the future, with the availability of more and more images, representing diverse cases, the efficiency of the models may be scaled up.

## Figures and Tables

**Table 1:** Division of original images into training and external validation datasets.

Sr. No.	Dataset type	Original chest X-ray images	Training dataset images (90%)	External validation dataset-I images (10%)	External validation dataset-II images (newly available images on COVID-19 chest X-ray image database from 19 March 2020 to 29 May 2020)
1	COVID-19	51	46	5	75
2	Non-COVID-19	21	19	2	N/A
3	Pneumonia	160	144	16	N/A
4	TB (Montgomery County X-ray Set)	54	49	5	N/A
5	Normal (Montgomery County X-ray Set)	66	59	7	N/A
	Total images	352	317	35	75

**Table:** (a) Original images/single augmentation-based models (for 26 models)

Sr. No.	Hyperparameter	Value
1	Number of iterations	400
2	Batch size	16
3	Number of epochs	24
4	Image size	256
5	Internal validation size	0.1
6	Filter size (1st convolutional layer)	3
7	Number of filters (1st convolutional layer)	32
8	Filter size (2nd convolutional layer)	5
9	Number of filters (2nd convolutional layer)	64
10	Filter size (3rd convolutional layer)	7
11	Number of filters (3rd convolutional layer)	128
12	Fully connected layer size	256

**Table:** (b) Original and all augmentation-based models (for combined image-based models)

Sr. No.	Hyperparameter	Value
1	Number of iterations	5568/11136/23300
2	Batch size	32
3	Number of epochs	24/49/101
4	Other hyperparameters	Same as given in (a)

**Table 3:** Performance of augmented image-based models on training, internal validation, and external validation images.

Results of the training and internal validation (*N* = 317)				Results of the external validation (*N* = 110)					
Augmentation type	Training accuracy (*N* = 286)	Validation accuracy (*N* = 31)	Validation loss	Normal (*N* = 7)	COVID-19 (*N* = 5)	New COVID-19 (*N* = 75)	Non-COVID-19 (*N* = 2)	Pneumonia (*N* = 16)	Tuberculosis (*N* = 5)
Rotate 120°	100	62	1.49	14.2	20	14.67	100	100	100
Rotate 140°	100	81.2	0.74	100	100	94.67	0	93.75	0

**Table 4:** Performance of combined models on training, internal validation, and external validation images.

Sr. No.	Name of the model	Results of the internal validation			Results of the external validation (using all test set images combined)					
	Model type	Training accuracy (*N* = 7418)	Validation accuracy (*N* = 824)	Validation loss	Normal (*N* = 182)	COVID-19 (*N* = 130)	New COVID-19 (*N* = 1950)	Non-COVID-19 (*N* = 52)	Pneumonia (*N* = 416)	TB (*N* = 130)
1	Combined model 1 (24 epochs)	100	93.8	0.126	73.63	56.92	42.62	53.85	96.39	75.38
2	Combined model 2 (49 epochs)	100	100	0.015	86.26	65.38	43.59	42.31	96.15	73.08
3	Combined model 3 (101 epochs)	100	93.8	0.524	85.71	70.77	51.28	51.92	93.99	74.62

**Table 5:** Performance of combined models on the testing set of 60° rotated images.

Augmentation type	Combined model 1 (24 epochs)						Combined model 2 (49 epochs)						Combined model 3 (101 epochs)					
	N^∗^	C^∗^	C^∗∗^	N-C^∗^	P^∗^	TB^∗^	N^∗^	C^∗^	C^∗∗^	N-C^∗^	P^∗^	TB^∗^	N^∗^	C^∗^	C^∗∗^	N-C^∗^	P^∗^	TB^∗^
Rotate 60°	85.71	80	52	100	100	80	100	80	65.33	100	100	60	100	100	66.67	100	93.75	80

N^∗^ = normal (*n* = 7); C^∗^ = COVID-19 (*n* = 5); C^∗∗^ = new COVID-19 (*n* = 75); N-C^∗^ = non-COVID-19 (*n* = 2); P^∗^ = pneumonia (16); TB^∗^ = tuberculosis (*n* = 5).

## Data Availability

The codes used for the development and validation of AI-based prediction models, including sample images, developed models, and scripts used for augmentation of the original images, have been uploaded on GitHub at https://github.com/arunsharma8osdd/covidpred.

## Supplementary Materials

Supplementary 1 Consists of all the supplementary tables (Tables S1-S9).

Supplementary 2 Consists of all the supplementary figures (Figures S1-S3).
