# Supplementary material for: Artificial Intelligence-Based Classification of Chest X-Ray Images into COVID-19 and Other Infectious Diseases
Source: Int J Biomed Imaging. 2020 Oct 6;2020:8889023. doi: 10.1155/2020/8889023 (PMC7539085; doi:10.1155/2020/8889023)
Supplement: Supplementary 1 — Consists of all the supplementary tables (Tables S1-S9). [file 8889023.f1.docx]

**Supplementary Figures**

**Artificial-Intelligence Based Classification of Chest X-ray Images into COVID-19 and Other Infectious Diseases**

Arun Sharma,^1#^ Sheeba Rani,^1#^ Dinesh Gupta^1^

^1^Translational Bioinformatics Group, International Centre for Genetic Engineering and Biotechnology (ICGEB), Aruna Asaf Ali Marg, New Delhi-110067, India.

^#^Arun Sharma and Sheeba Rani contributed equally to this work.

Correspondence should be addressed to Dinesh Gupta; dinesh@icgeb.res.in

**
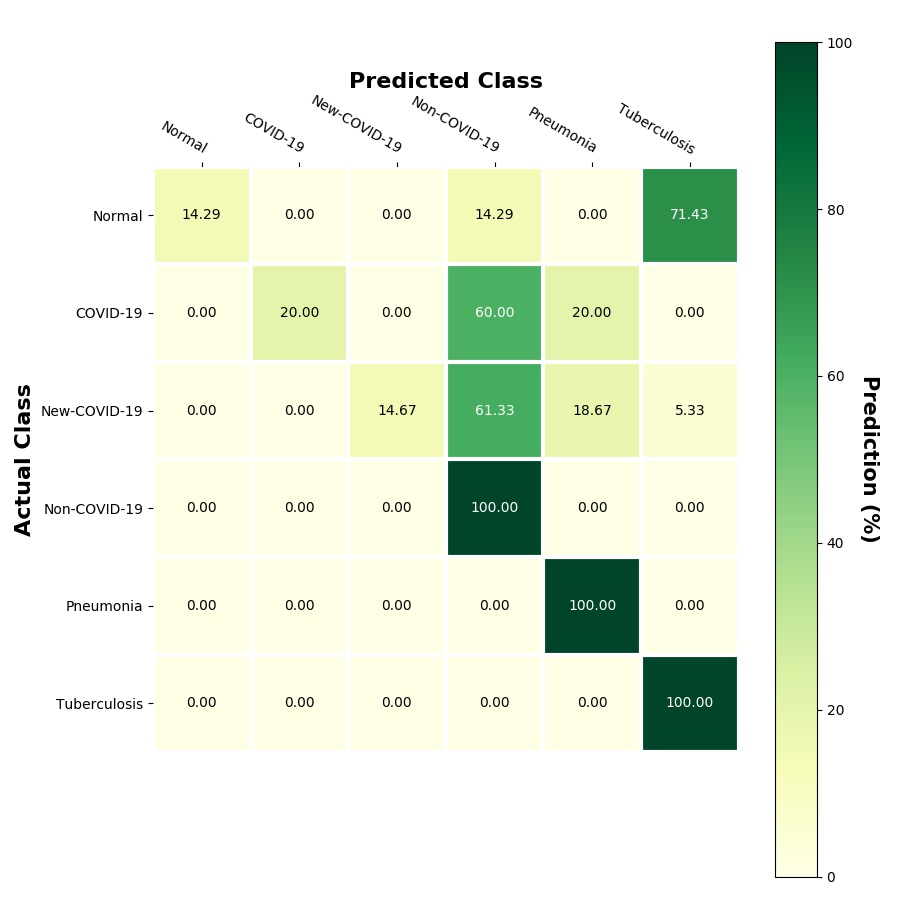
**

Figure S1: The results of the evaluation of rotate 120° images based model with external validation dataset-I and II (used 120° rotated images only).

**
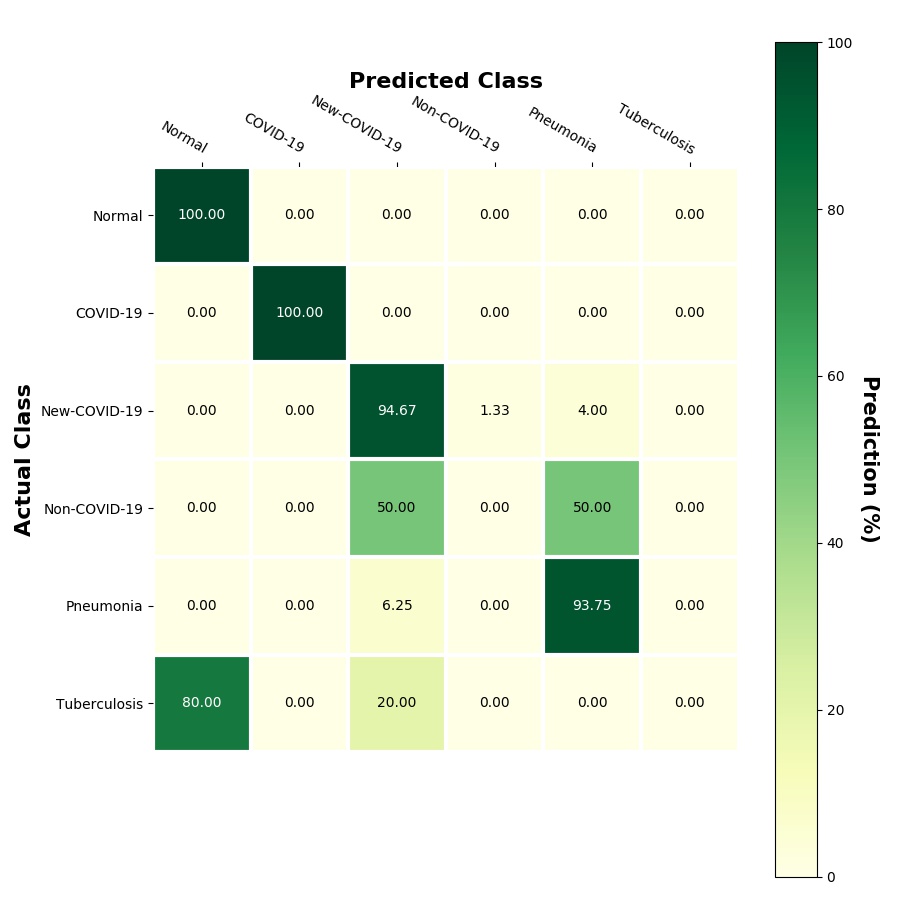
**

Figure S2: The results of the evaluation of rotate 140° images based model with external validation dataset-I and II (used 140° rotated images only).

**
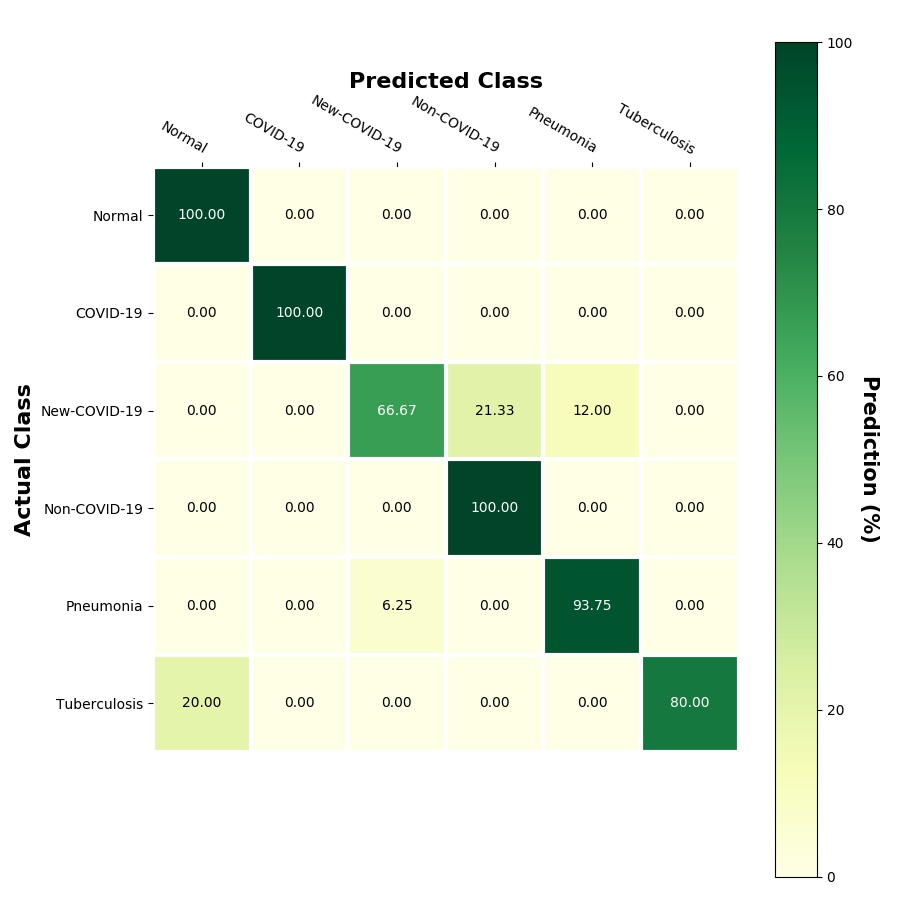
**

Figure S3: The results of the evaluation of combined model 3 (101 epochs based) with external validation dataset-I and II (used 60° rotated images only).
